# Phylodynamics of dengue virus 2 in Nicaragua leading up to the 2019 epidemic reveals a role for lineage turnover

**DOI:** 10.1186/s12862-023-02156-4

**Published:** 2023-09-28

**Authors:** Panpim Thongsripong, Sean V. Edgerton, Sandra Bos, Saira Saborío, Guillermina Kuan, Angel Balmaseda, Eva Harris, Shannon N. Bennett

**Affiliations:** 1 Florida Medical Entomology Laboratory, Institute of Food and Agricultural Sciences, University of Florida, Vero Beach, FL USA; 2 Interdisciplinary Studies Graduate Program, The University of British Columbia, Vancouver, BC Canada; 3 Division of Infectious Diseases and Vaccinology, School of Public Health, University of California, Berkeley, Berkeley, CA USA; 4 Centro Nacional de Diagnóstico y Referencia, Laboraorio Nacional de Virología, Ministry of Health, Managua, Nicaragua; 5 Sustainable Sciences Institute, Managua, Nicaragua; 6 Centro de Salud Sócrates Flores Vivas, Ministry of Health, Managua, Nicaragua; 7 Department of Microbiology, California Academy of Sciences, San Francisco, CA USA

**Keywords:** Dengue virus, Virus evolution, Lineage turnover, Clade replacement, Natural selection, Phylodynamics, Nicaragua

## Abstract

**Background:**

Dengue is a mosquito-borne viral disease posing a significant threat to public health. Dengue virus (DENV) evolution is often characterized by lineage turnover, which, along with ecological and immunological factors, has been linked to changes in dengue phenotype affecting epidemic dynamics. Utilizing epidemiologic and virologic data from long-term population-based studies (the Nicaraguan Pediatric Dengue Cohort Study and Nicaraguan Dengue Hospital-based Study), we describe a lineage turnover of DENV serotype 2 (DENV-2) prior to a large dengue epidemic in 2019. Prior to this epidemic, Nicaragua had experienced relatively low levels of DENV transmission from 2014 to 2019, a period dominated by chikungunya in 2014/15 and Zika in 2016.

**Results:**

Our phylogenetic analyses confirmed that all Nicaraguan DENV-2 isolates from 2018 to 2019 formed their own clade within the Nicaraguan lineage of the Asian/American genotype. The emergence of the new DENV-2 lineage reflects a replacement of the formerly dominant clade presiding from 2005 to 2009, a lineage turnover marked by several shared derived amino acid substitutions throughout the genome. To elucidate evolutionary drivers of lineage turnover, we performed selection pressure analysis and reconstructed the demographic history of DENV-2. We found evidence of adaptive evolution by natural selection at the codon level as well as in branch formation.

**Conclusions:**

The timing of its emergence, along with a statistical signal of adaptive evolution and distinctive amino acid substitutions, the latest in the NS5 gene, suggest that this lineage may have increased fitness relative to the prior dominant DENV-2 strains. This may have contributed to the intensity of the 2019 DENV-2 epidemic, in addition to previously identified immunological factors associated with pre-existing Zika virus immunity.

**Supplementary Information:**

The online version contains supplementary material available at 10.1186/s12862-023-02156-4.

## Introduction

Dengue is a mosquito-borne viral disease whose ongoing global expansion poses a significant threat to public health [1]. Since the discovery of the causative agent in 1943 [2], the impacts of dengue virus (DENV) have played out at different scales, from sporadic regional outbreaks by locally restricted strains throughout the mid-20th century, to the global circulation of multiple strains and serotypes such that dengue is now endemic in over 100 countries and threatens 40% of the world’s population [3]. A recent study estimated that 100 million dengue infections occurred in 2017, increasing from 23 million dengue infections in 1990, with potential for further spread [4].

Dengue is caused by one of the four DENV serotypes (DENV-1 to -4) in the genus *Flavivirus*, family *Flaviviridae*. Each serotype consists of various genotypes, which in turn can be further subdivided into clades [5]. The DENV genome is approximately 10.7 kb in length and comprised of a positive-sense RNA strand that encodes three structural (C, prM/M, and E) and seven nonstructural proteins (NS1, NS2A, NS2B, NS3, NS4A, and NS5) [6]. Viral infection leads to a range of disease manifestations, in which the majority of cases are either asymptomatic or present as a nonspecific febrile illness [7]. Classic dengue fever (DF) or “breakbone fever” can progress to dengue haemorrhagic fever/dengue shock syndrome (DHF/DSS), with manifestations such as hemorrhage and hypovolemic shock, that can be life-threatening and that is becoming more common in many parts of the world [4, 8–12].

DENV evolution is often characterized by ongoing evolution within circulating lineages over time interspersed with occasional lineage turnover, in which a new lineage of viruses replaces a former one [13–19]. The resulting pattern forms a ladderlike tree topology until said lineage disappears and a new and often very distinct clade accounts for ongoing cases [17]. Although lineage replacement is often associated with the introduction of viruses from other populations, in contrast to ongoing within-lineage evolution occurring in situ, nonetheless there are times when lineage turnover is not associated with a detectable introduction. Major clade replacements, along with ecological and immunological factors [20], have been linked to changes in dengue phenotype including infectivity, severity, and others potentially related to epidemic dynamics [9, 21–24]. Thus, other mechanisms of lineage turnover besides the movement of virus lineages between distinct populations or countries are worth exploring.

The evolutionary mechanisms underlying major clade replacement include mutation arising in situ or through introductions followed by selection or genetic drift. Selection can then operate on standing genetic diversity to increase the frequency of the fittest variant, or decrease the frequency of the less fit ones, leading to their fixation or replacement, respectively. In contrast, genetic drift describes the random fixation of a variant, resulting in stochastic fluctuations in variant frequencies over time, independent of their fitness or phenotype, although phenotypic impacts can be observed. Previous studies show that both selection and drift are important forces that shape DENV clade replacement [17, 22, 25–28].

**Fig. 1:**
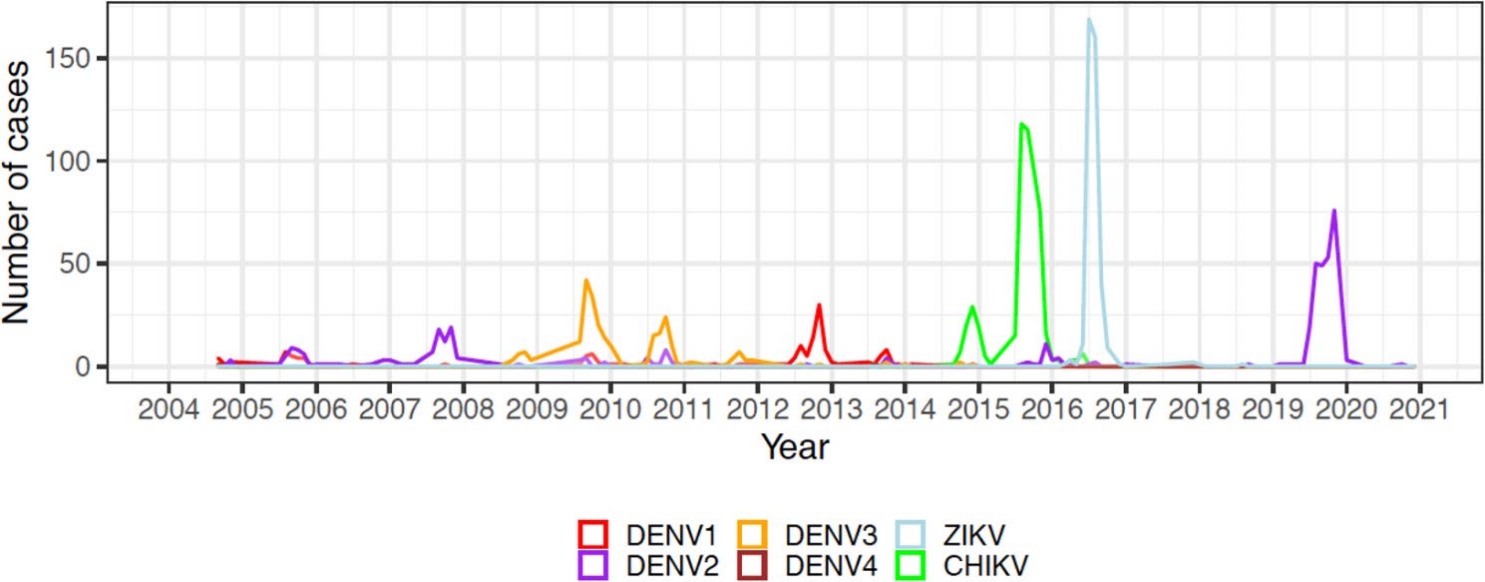
Confirmed arboviral cases caused by DENV, CHIKV or ZIKV in Nicaragua, 2004–2021

Here, we describe a lineage turnover event in DENV-2 following a period of low DENV activity, culminating in the emergence of a highly distinct lineage leading up to the largest dengue epidemic in Nicaragua’s recorded history in 2019/20 (Fig. 1). Prior to this epidemic, DENV transmission had largely been replaced by epidemics of chikungunya virus (CHIKV, 2014/15) and Zika virus (ZIKV, 2016), and interestingly, increases in dengue disease severity has been associated with pre-existing ZIKV immunity [20]. Two unique long-term population-based studies (the Nicaraguan Pediatric Dengue Cohort Study, or PDCS, and the Nicaraguan Dengue Hospital-based Study) based in the capital city of Managua provided valuable epidemiologic, clinical, and virologic data [29–31]. In addition, we obtained samples from León, a city in western Nicaragua that experienced an increase in DENV transmission starting in 2018 associated with increased rates of severe dengue. Our objectives were to identify the origins of the DENV strain(s) associated with the 2018 outbreak in León through to the 2019 epidemic in Managua and beyond, and to elucidate the evolutionary mechanisms of intensified epidemic activity and lineage turnover. We reconstructed the phylogenetic history of DENV-2 based on whole genome genetic characterization and performed selection pressure analysis on the coding gene region based on non-synonymous substitution rates over the period of study. We also reconstructed the demographic history of DENV-2 based on genetic diversity and phylodynamics. Overall, our study illustrated another instance of DENV lineage turnover during intensified DENV transmission in Nicaragua against the backdrop of changing flaviviral dynamics, such as recent ZIKV activity and resultant changes in the immunological landscape [20]. Our study provides insights into the relative importance of different evolutionary mechanisms driving DENV-2 epidemics in the region.

## Results

### DENV-2 phylogeny

**Fig. 2:**
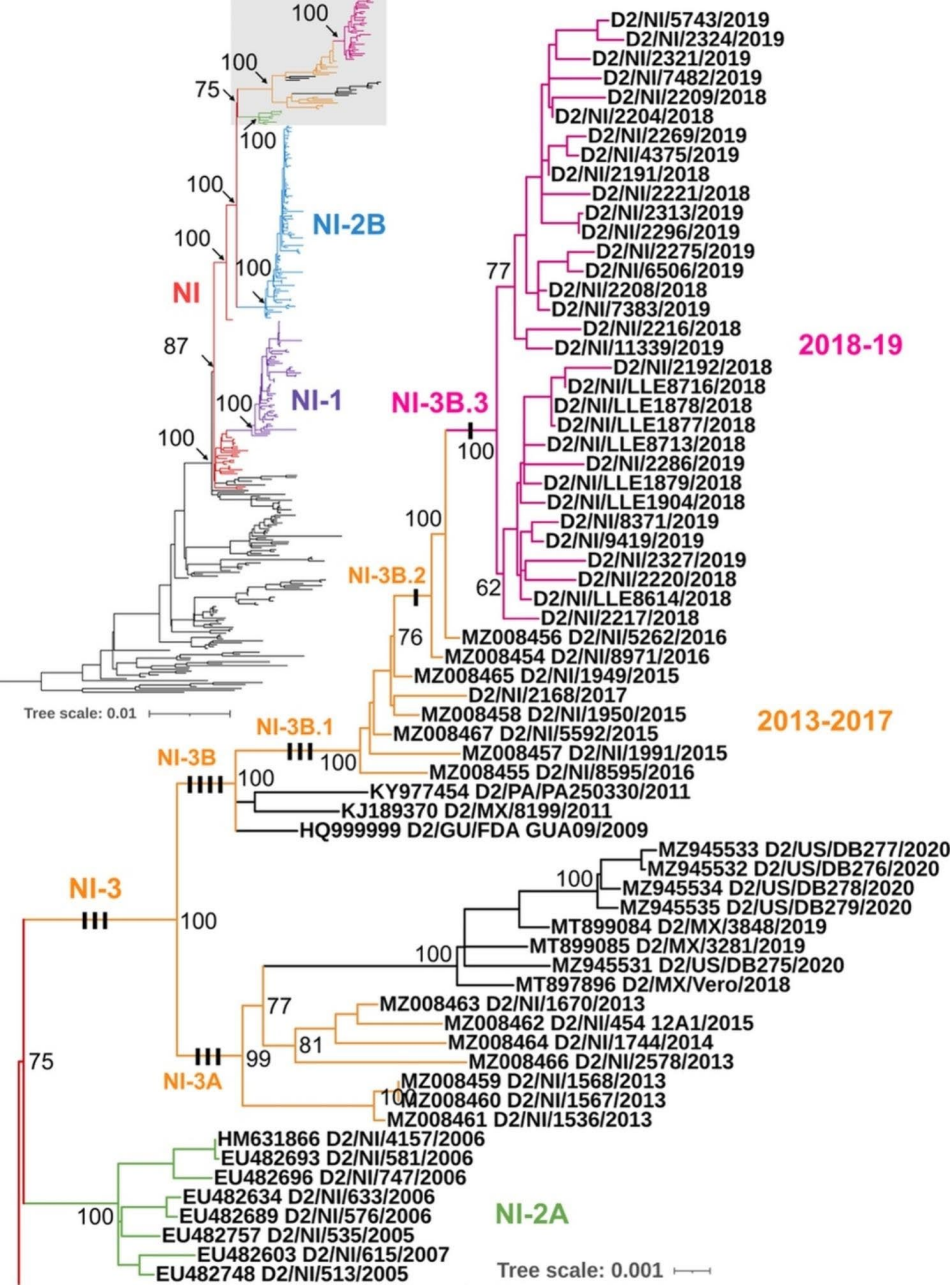
Maximum likelihood (ML) phylogeny of DENV-2. Thirty-two newly sequenced genomes from Managua and León, Nicaragua, are shown in the clade colored magenta (samples from León are denoted by “LLE”). The enlarged tree on the right represents the phylogeny shaded gray in the tree in the upper left-hand corner. Sequences are labeled by ISO country code, sample identifier, and year of isolation. Bootstrap values are shown for each clade. Each black bar on the branches represents a clade-specific non-synonymous mutation

Dengue virus (DENV) low-passage cell culture supernatant derived from 32 serum/plasma samples from the Nicaraguan PDCS study, Dengue Hospital-based study, and the city of Léon collected between September 2018 and August 2019 were sequenced. To determine the evolutionary relationships among DENV-2, we combined the newly sequenced genomes with other publicly available sequence data (a total of 358 DENV-2 genomic sequences), and inferred phylogenetic trees using maximum likelihood (ML) and Bayesian Markov chain Monte Carlo methods (the latter resulting in a Maximum Clade Credibility, or MCC, tree). The topology of the ML tree (Fig. 2) is in congruence with the MCC tree (Supplementary Fig. 1). All Nicaraguan DENV-2 isolates formed a distinct clade (called Nicaraguan clade, or “NI” [31]) within the Asian/American genotype. Within the NI clade, DENV-2 sampled since the 2013/14 transmission season formed a distinct clade that includes samples from as early as 2015 up until 2019. Prior to this most recent lineage, a previous study identified 2 subclades: NI-1, which comprised a majority of Nicaraguan DENV-2 isolated in the 2004/2005 and 2005/2006 seasons, and NI-2, which replaced NI-1 after the 2005/2006 seasons [31]. NI-2 was further subdivided into NI-2 A and NI-2B. NI-2 A contained a relatively small number of sequences from the 2005/2006 and 2006/2007 seasons, while NI-2B rapidly became dominant in the country, containing a majority of sequences from the 2006/2007 to 2008/2009 seasons. Our phylogenetic analyses indicated that all 2018 and 2019 samples sequenced in this study, along with strains isolated in Nicaragua since 2013, formed a clade that is closely related to and likely descended from the NI-2 A clade, which we designate as NI-3 (Fig. 2 and Supplementary Fig. 1), and which in turn emerged in association with increased DENV-2 activity in 2016 [32].

This new clade NI-3, is further comprised of two well-supported subclades: (1) NI-3 A contains several Nicaraguan sequences isolated between 2013 and 2015, as well as multiple sequences from Mexico and the US (imported cases from Mexico) isolated between 2018 and 2020; and (2) NI-3B comprises a majority of DENV-2 Nicaraguan sequences collected since 2015, including isolates from 2018 to 2019 that formed their own clade (referred to as NI-3B.3, Fig. 2). Although samples were derived from various localities in the country, the León samples clustered together non-exclusively and with weaker support (ML bootstrap support of 62), suggestive of some geographic structure in the distribution of DENV-2. Interestingly, DENV-2 sequences from other countries (Mexico, Guatemala, and Panama) isolated many years prior fell basal to the NI-3B clade (Fig. 2), suggesting that the ancestral viruses to NI-3B were widespread, that is at least in three countries, across Central America.

Based on the Bayesian inference, the evolutionary rate of the sampled DENV-2 population was estimated to be 7.97 × 10^− 4^ substitutions/site/year with 95% highest posterior density (HPD) interval of [7.38 × 10^− 4^, 8.57 × 10^− 4^]. Our analysis (Supplementary Fig. 1) indicates that the NI clade emerged as early as 1996 (22.02 years before 2019, with 95% HPD of [22.76, 21.41]). The most recent common ancestor (MRCA) of the NI-3B.3 clade was estimated to have existed in late 2016 (2.29 years before 2019; 95% HPD of [2.76, 1.87]). The MRCA of the NI-3 clade existed by early 2006 (12.77 years before 2019; 95% HPD of [14.11, 11.72]). The MRCA of the NI-3 and NI-2 A clades existed by late 2000 (18.1 years before 2019; 95% HPD of [19.27, 16.88]).

### Clade-specific non-synonymous mutations

**Fig. 3:**
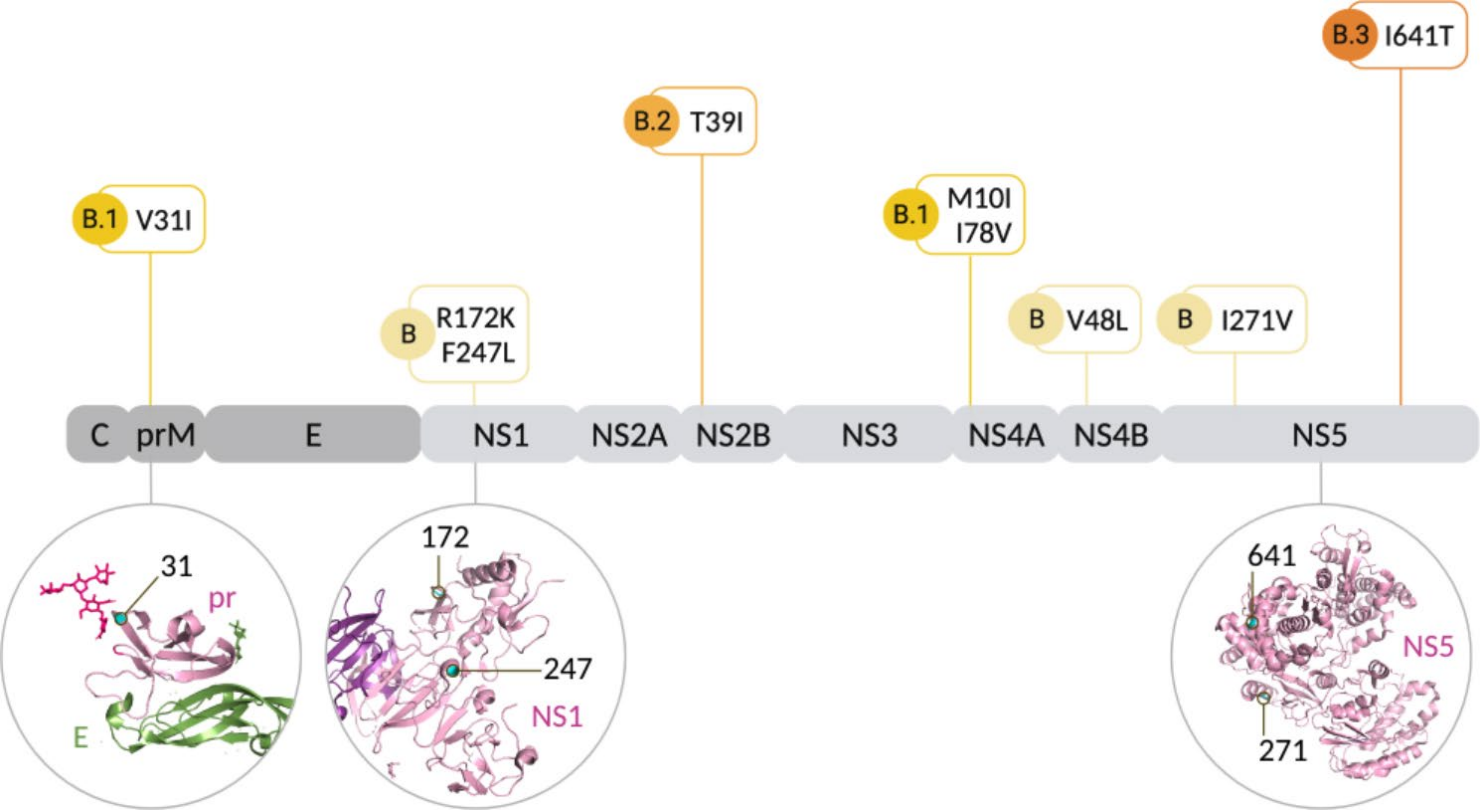
Locations of non-synonymous mutations (cyan) associated with the evolution of NI-3B clade on a DENV-2 genome, with pr-E, NS1, and NS5 protein structures

We identified 15 non-synonymous mutations associated with the evolution of NI-3 clades when compared to immediately ancestral Nicaraguan clades (Fig. 2; Table 1). These mutations occur in both structural (prM and E) and nonstructural genes (NS1, NS2A, NS2B, NS4A, NS4B, and NS5). Out of 15 amino acid substitutions observed, 5 resulted in hydropathy and polarity changes and 2 in charge changes (Table 1). These non-conservative substitutions were found in the E protein (M118K and T200I, shared derived substitutions of clade NI-3 A), NS2A (N139K, a shared derived substitution of clade NI-3 A), NS2B (T39I, a shared derived substitution of clade NI-3B.2), NS4A (M10I, a shared derived substitution of clade NI-3B.1), and NS5 (I641T, a shared derived substitution of clade NI-3B.3, the new clade identified in this study). Of 15 substitutions, nine substitutions accumulated as unique synapomorphies to the various sublineages evolving within the branch of NI-3, starting with four founder substitutions occurring in the initial formation of NI-3B (Fig. 3). These nine substitutions occurred in several different genes: prM, NS1, NS2B, NS4A, NS4B, and NS5.

**Table 1:** Non-synonymous mutations associated with Nicaraguan DENV-2 clades

Gene	Position	NI	NI-3	NI-3 A	NI-3B	NI-3B.1	NI-3B.2	NI-3B.3	Changes in hydropathy	Changes in charge	Changes in polarity
		(1999- 2002)	(2013- 2019)	(2013- 2015	(2015- 2019)	(2015- 2019)	(2016- 2019)	(2018- 2019)			
**prM**	31	V (GTT)	V (GTT)	V (GTT)	V (GTT)	**I (ATT)**	**I (ATT)**	**I (ATT)**	None	None	None
**E**	118	M (ATG)	M (ATG)	**K (AAG)**	M (ATG)	M (ATG)	M (ATG)	M (ATG)	Hydrophobic to Hydrophilic	Uncharged to Positively charged	Nonpolar to Polar
**E**	200	T (ACA)	T (ACA)	**I (ATA)**	T (ACA)	T (ACA)	T (ACA)	T (ACA)	Neutral to Hydrophobic	None	Polar to Nonpolar
**NS1**	172	R (AGA)	R (AGA)	R (AGA)	**K (AAA)**	**K (AAA)**	**K (AAA)**	**K (AAA)**	None	None	None
**NS1**	247	F (TTT)	F (TTT)	F (TTT)	**L (TTA)**	**L (TTA)**	**L (TTA)**	**L (TTA)**	None	None	None
**NS2A**	136	I (ATA)	**V (GTA)**	**V (GTA)**	**V (GTG)**	**V (GTG)**	**V (GTG)**	**V (GTG)**	None	None	None
**NS2A**	139	N (AAT)	N (AAT)	**K (AAA)**	N (AAT)	N (AAT)	N (AAT)	N (AAT)	None	Uncharged to Positively charged	None
**NS2B**	39	T (ACC)	T (ACC)	T (ACC)	T (ACC)	T (ACC)	**I (ATC)**	**I (ATC)**	Neutral to Hydrophobic	None	Polar to Nonpolar
**NS4A**	10	M (ATG)	M (ATG)	M (ATG)	M (ATG)	**I (ATA)**	**I (ATA)**	**I (ATA)**	Neutral to Hydrophobic	None	Polar to Nonpolar
**NS4A**	78	I (ATA)	I (ATA)	I (ATA)	I (ATA)	**V (GTA)**	**V (GTA)**	**V (GTA)**	None	None	None
**NS4B**	48	V (GTC)	V (GTC)	V (GTC)	**L (CTC)**	**L (CTC)**	**L (CTC)**	**L (CTC)**	None	None	None
**NS4B**	156	E (GAA)	**D (GAC)**	**D (GAC)**	**D (GAC)**	**D (GAC)**	**D (GAC)**	**D (GAC)**	None	None	None
**NS5**	271	I (ATA)	I (ATA)	I (ATA)	**V (GTA)**	**V (GTA)**	**V (GTA)**	**V (GTA)**	None	None	None
**NS5**	521	D (GAC)	**E (GAA)**	**E (GAA)**	**E (GAA)**	**E (GAA)**	**E (GAA)**	**E (GAA)**	None	None	None
**NS5**	641	I (ATC, ATT*)	I (ATC)	I (ATC)	I (ATC)	I (ATC)	I (ATC)	**T (ACC)**	Hydrophobic to Neutral	None	Nonpolar to Polar

### Selection pressure analysis

Selection pressure analysis of the complete coding region of DENV-2 was performed using a combination of tests (Branch-Site Unrestricted Statistical Test for Episodic Diversification; BUSTED, Adaptive Branch-Site Random Effects Likelihood; aBSREL, and Mixed Effects Model of Evolution; MEME) available in HyPhy package as implemented in Datamonkey server. BUSTED, incorporating synonymous rate variation, found evidence (LRT, p-value = ≤ 0.05) of gene-wide episodic diversifying selection across our Nicaragua-focused phylogeny (Supplementary Fig. 1, gray shaded area). Gene-wide dN/dS distribution identified a category of structural and non-structural sites under strong positive selection (estimates for categories of the omega variable ω representing dN/dS were as follows: ω_1_ = 0.07 (99.15%), ω_2_ = 0.07 (0.84%), ω_3_ = 941.49 (0.01%)). aBSREL found evidence of episodic diversifying selection on 6 of 541 branches in the DENV-2 phylogeny. These 6 branches are all terminal branches. The ω distribution over sites at each significant branch and the inferred aBSREL model complexity are summarized in Supplementary Tables 1 and 2, respectively. MEME found evidence of episodic positive selection at 16 sites below the p-value threshold of 0.05 (Table 2). Of the 16 positively selected sites, 2 sites were located in a structural gene (E), while the remaining 14 sites were located in non-structural genes (NS1, NS2A, NS2B, NS3, NS4B, and NS5 genes).

**Table 2:** Sites that experienced episodic positive selection, identified using the MEME test

Site	alpha	beta-	beta+	p-value	Number of branches under selection	Protein	Position in protein
620	0	0	208.26	< 0.01	1	E	340
643	0.42	0.15	252.91	< 0.01	3	E	363
982	0.27	0.1	576.29	< 0.01	2	NS1	207
1005	0	0	1026.38	< 0.01	1	NS1	230
1407	0	0	10,000	< 0.01	1	NS2A	72
1860	0	0	253.18	< 0.01	1	NS2A	119
2399	0.66	0	10,000	< 0.01	3	NS2B	62
2487	0.11	0.02	10,000	< 0.01	1	NS2B	92
2610	0	0	902.92	< 0.01	1	NS3	15
1199	0	0	63.51	0.01	2	NS3	384
1246	0	0	341.74	0.01	4	NS3	385
1438	1.22	1.22	1009.51	0.01	1	NS4B	96
1859	0.92	0.19	4896.97	0.01	1	NS4B	244
1489	0	0	3.14	0.03	5	NS5	119
2659	1.03	0.28	196.38	0.03	2	NS5	168
3371	0	0	19.33	0.04	2	NS5	380

### Reconstruction of demographic history

**Fig. 4:**
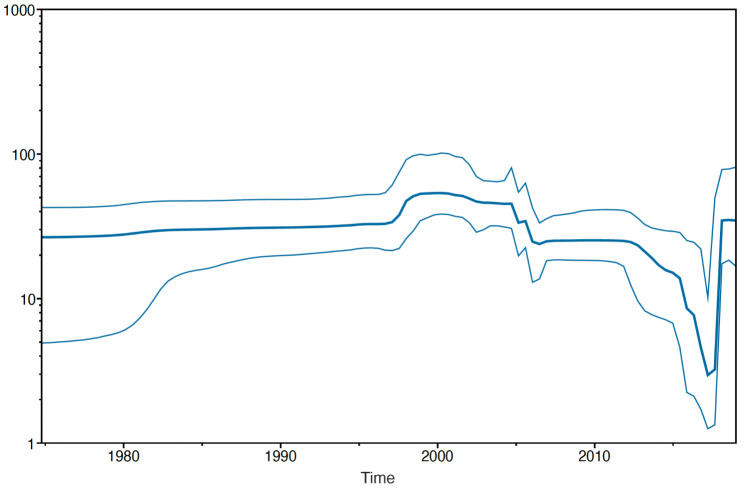
Bayesian skyline plot showing the relative effective population size of DENV-2 infections in the sampled population. The y-axis represents the effective number of infections in log_10_ scale. The x-axis represents the year. The thick blue line represents the median estimate, and the credibility interval based on 95% HPD interval is represented by thin blue lines

To examine the demographic history of the sampled DENV-2 population from our study region over time, the relative effective population size (N_e_) was inferred from dynamics in genetic diversity using the Bayesian skyline model. As shown in Fig. 4, N_e_ appeared to be steady until 1997, when a sharp increase was observed, resulting in the first peak of N_e_ that lasted from 1997 to 2004. This was followed by a sharp decline resulting in the lowest estimates of population size based on diversity in 2016. Correspondingly, Nicaragua experienced a massive ZIKV epidemic in 2016 following its introduction into the region, with generally low to undetected DENV transmission during this period (Fig. 1). The second sharp increase in population size, reaching a level almost as high as the first peak, was observed in 2018 and 2019, during the large DENV2 epidemic.

## Discussion

Our study combined newly sequenced DENV-2 genomes from two ongoing Nicaraguan cohort and hospital studies initiated in 2004 and 2005, respectively, with publicly available genomes to identify the potential origins and evolutionary drivers of a severe DENV-2 epidemic peaking in 2019 in Nicaragua. This epidemic was presaged by a notable number of severe cases in the city of León in western Nicaragua starting in 2018. Our phylogenetic analysis confirmed that these Nicaraguan DENV-2 isolates, collected between September 2018 and August 2019, formed a single clade (NI-3B.3) within the Nicaraguan lineage (NI) of the Asian/American genotype. Importantly, the emergence of the NI-3B.3 lineage and its rise to dominance associated with the 2019 epidemic reflects a DENV-2 lineage turnover event in which the formerly dominant NI-2B clade presiding from 2005 to 2009 [31] was replaced by this new and potentially more fit clade (NI-3) containing DENV-2 isolates sequenced in this study.

Based on its relationship to sequences from the region, this NI-3 clade likely evolved within Nicaragua, rather than elsewhere coupled with importation, and is more closely related to NI-2 A (bootstrap support of 75, and posterior support of 84), a small clade that co-circulated along with NI-2B between 2005 and 2007 in Nicaragua [31]. We acknowledge that a general lack of sequence-based surveillance in the region means that there is an under-representation in our dataset of foreign samples relative to Nicaraguan samples. This sampling bias limits our ability to identify potential foreign sources and importation/exportation events. Our analyses indicated that the MRCA of the NI-3 and NI-2 A clade existed in late 2000, not long after the NI-2B clade emerged (Supplementary Fig. 1). This period (2000) coincided with a high effective population size for DENV-2, reflecting increased genetic diversity within the virus population that in turn may have provided the genetic innovations that gave rise to the highly successful NI-3 lineage.

Multiple hypotheses for lineage turnover or clade replacement in DENV have been proposed. First, stochastic events may result in a random sampling of viral variants, especially during low transmission years [22]. Second, genotypes with higher replicative fitness in either the human host [25, 26] or the mosquito vector [17, 27, 28] could have increased transmissibility and potentially replace less fit clades. In fact, increased replicative fitness of DENV-2 in native mosquitoes contributed to the replacement of NI-1 by the NI-2B clade [27]. Finally, selection pressures, such as prior host immunity, can lead to changes in the fitness landscape in which some viruses become fitter than others (e.g., those that can evade host immune responses and replicate effectively), resulting in another example of adaptive evolution [33]. In reality, these forces are difficult to tease apart and likely combine to create conditions that favor one strain over another, and potentially drive the observed non-synonymous mutations to fixation [27, 31].

Interestingly, we found evidence of adaptive evolution by natural selection at the codon level as well as in branch formation of our DENV sequences and the NI-3B.3 clade, whose emergence coincided with the intensification of DENV disease incidence and severity in 2018-19. Although, no association between the sites that experienced episodic positive selection identified using the MEME test and the non-synonymous substitutions has been found. Prior to this epidemic, Nicaragua had experienced relatively low levels of DENV transmission since the 2013/14 season. This period included the introduction of chikungunya virus (CHIKV) into the region in 2014 followed by ZIKV in 2016, which resulted in a massive epidemic. Interestingly, a previous study utilizing epidemiological and immunological data from the same cohorts demonstrated that prior ZIKV infection and low to intermediate levels of anti-DENV antibodies post-ZIKV increased symptomatic infections and enhanced disease severity in the subsequent DENV-2 epidemics in 2019 [20]. The timing of the emergence of NI-3B.3 with this epidemic, along with a statistical signal of adaptive evolution and a distinctive mutation in the NS5 gene unique to this lineage (non-synonymous, non-conservative mutation I641T), leads us to propose that this lineage may have increased fitness relative to prior strains and may have contributed to the increase in size and severity of the 2018/19 DENV-2 epidemic, in addition to the previously identified immunological factors [20]. However, we do not have further information, nor is it within the scope of this study, to determine the importance of this mutation in terms of virus phenotype and the mechanisms by which the mutation might increase viral fitness and/or epidemic severity. Further laboratory experiments are needed to confirm this hypothesis.

Further experiments on potential phenotypic mechanisms underlying adaptive evolution would be best guided by the functional role of NS5, as the largest and the most conserved flaviviral protein (with ~ 70% sequence identity across the four DENV serotypes [34]), with a methyltransferase domain (MTase) responsible for capping the viral RNA, and an RNA-dependent RNA polymerase (RdRp), which replicates viral RNA [35]. In addition to its role in genome replication, NS5 proteins also play a crucial part in evading the host immune system [35]. The amino acid residue 641 is located within the RdRp domain of the NS5. Although DENV lineage turnover events have been observed in multiple studies, key viral determinants of increased fitness have not been consistently detected. Previously observed lineage turnover events were associated with signals of positive selection in Capsid [18], NS1 [19], NS2A [13, 15], and NS5 [18]. This is not surprising given that the underlying mechanisms driving positive selection and the selective pressures acting on DENV genomes likely varied considerably among settings.

Genetic drift is also frequently evaluated for its potential role in lineage turnover by estimating the effective population size (N_e_): when N_e_ is small, sampling effects are magnified between generations, and allele frequencies can fluctuate strongly [36]. Our analysis indicated that N_e_ of DENV-2 decreased during 2014–2017, and thus may have facilitated random genetic drift leading to the fixation of NI-3B.3 during this period, regardless of any phenotypic fitness-related considerations. Thus, we cannot rule out the role of multiple mechanisms underlying the turnover of DENV-2 lineages leading to the emergence and dominance of NI-3B.3. Likely, genetic drift early in the origin of NI-3B.3, combined with natural selection further promoting the fixation of underlying mutations led to subsequent dominance of the NI-3B.3 clade.

Our study is limited by heterogeneous sampling of DENV-2 isolates in other countries, compared to the more systemic sampling in Nicaragua, where samples are collected over time and randomly subsampled for sequencing for multi-year representation in our phylogenies. This systematic approach has resulted in well-supported and reproducible phylogenies of the evolutionary dynamics within Nicaragua, including the relative placement of foreign samples in spite of their under-representation in the overall tree. Specifically, in our phylogenetic analysis, we observed DENV-2 strains from Mexico, Panama, and Guatemala (isolated in 2011, 2011, and 2009, respectively) which were robustly related to, but distinct from our NI-3B clade. However, because of the limited sampling in these neighboring countries over time, it is impossible to draw conclusions about the direction of virus movements. Furthermore, under-sampling throughout the region limits our power to detect potential importations of DENV-2 into the country.

## Conclusion

Utilizing existing samples and epidemiological data collected from the Nicaraguan pediatric hospital and cohort studies, along with a total-RNA sequencing approach, we documented a DENV-2 lineage turnover event during the 2018/19 dengue epidemic in Managua and León. This newly emerged lineage (NI-3) replaced the formerly dominant clade presiding from 2005 to 2009 (NI-2B). We found evidence of adaptive evolution by natural selection at the codon level as well as in branch formation of this new lineage. Although we cannot rule out other influencing mechanisms, such as genetic drift, that may play a role in the evolution of this new clade, the timing of its emergence, a statistical signal of natural selection, and a distinctive mutation in the NS5 gene led us to propose that this lineage may have increased fitness relative to prior strains, resulting in its fixation. Further laboratory-based studies are needed to test this hypothesis. Overall, our study revealed another example of DENV lineage turnover during an epidemic and shed light on possible mechanisms underlying this evolutionary phenomenon that have the potential to drive severe epidemiologic outcomes and impact global public health.

## Materials and methods

### Study population and DENV samples

Samples were collected as part of the ongoing Nicaraguan Pediatric Dengue Cohort Study (PDCS) and Dengue Hospital-based study, described in more detail in previous publications [29, 37]. The PDCS, established in 2004, followed ~ 3,800 children who agreed to seek medical care at the Health Center Sócrates Flores Vivas at the first sign of fever [29]. The Dengue Hospital-based Study, established in 2005, derived samples from patients with suspected dengue who presented to the Infectious Diseases Ward of the National Pediatric Reference Hospital, Hospital Infantil Manuel de Jesús Rivera, in Managua. Samples from León were obtained from the National Dengue Surveillance Program of the Nicaraguan Ministry of Health.

As part of the established protocol for the Nicaraguan PDCS and Hospital-based studies, all samples were screened for DENV, CHIKV(since 2014), and ZIKV (since 2015) by multiplex real-time RT-PCR [38, 39]. In the case of DENV-positive samples, serotype was assigned using serotype-specific RT-PCR [29, 40]. Dengue viruses from a subset of DENV-positive samples were isolated in C6/36 cells [41]. Virus passages in cell culture were restricted to one to two rounds to minimize selection in vitro.

In this particular study, 63 isolates of DENV-2 from late 2018 and early 2019 were sent to the California Academy of Sciences for sequencing. From these, 32 isolates with high DENV-2 titers were selected randomly, and their nearly complete genomes were successfully sequenced. These sequences represented samples collected between September 2018 and August 2019 and were from Nicaraguan PDCS and Hospital-based studies (Managua) or from the León population.

### RNA Extraction and cDNA synthesis

Steps for RNA extraction and cDNA synthesis followed closely to our previously described methods [32]. Briefly, 300–400 µl of cell culture supernatant was used for RNA extraction using the AgenCourt RNAdvance Cell V2 kit (Beckman Coulter Genomics, USA) according to the manufacturer’s protocols. First-strand cDNA was synthesized with random hexamers and the Superscript® III First-Strand Synthesis System (Thermofisher, USA). Second-strand cDNA synthesis was performed using Klenow exo-enzyme (New England Biolabs, USA). All cDNA samples were purified using AMPure XP (Beckman Coulter Life Sciences, USA).

### Library preparation and next-generation sequencing

Library preparation was performed using sparQ DNA Frag & Library Prep Kit (Quantabio, USA), according to the manufacturer’s instructions. Samples were indexed with molecular ID tags, which allowed for multiplex sequencing using sparQ adapters. Libraries were quantified and assessed for quality using an Agilent 2100 Bioanalyzer (Agilent Technologies, USA) and Qubit 2.0 Fluorometer (Life Technologies, USA). Samples were then pooled using equimolar pooling and quantified again using Qubit. Pooled library samples were loaded onto the Illumina MiSeq platform and sequenced in-house using paired-end, dual-indexed sequencing and 600-cycle MiSeq Reagent kit v3 (2 × 300 bp). No-template controls (NTCs) were included in all steps of laboratory processes and were quantified along with samples. We used a combination of cut-off values (Quantification cycle or C_q_ >30, and percentage of sequences mapped to DENV-2 less than what was found in the NTCs) to exclude samples from further analysis.

### Generation of consensus genomes

All sequence data analysis was performed in CLC Genomics Workbench 7.0.3 (https://www.qiagenbioinformatics.com/). First, reads were filtered for quality, and adaptors were trimmed using default parameters. High-quality reads were mapped using default parameters to reference genomes for each DENV serotype available in GenBank (NC_001477, DENV-1; NC_001474, DENV-2; NC_001475, DENV-3; and NC_002640, DENV-4). Virus consensus sequences were extracted using default parameters. These were then exported for phylogenetic analyses. Raw unprocessed sequencing reads are available through the NCBI Sequence Read Archive BioProject PRJNA970723. The assembled whole and partial virus genomes are available through the NCBI Nucleotide database (accession numbers OQ782198 - OQ782229).

### Phylogenetic analysis

Nicaraguan DENV-2 genomes from 2018/2019 were analyzed in a phylogenetic framework with other publicly available DENV-2 genomes. Phylogenetic analysis included both maximum-likelihood (ML) and Bayesian methods. An initial large comprehensive DENV-2 phylogeny was used to determine the genotype of the newly sequenced DENV-2 genomes and to explore their evolutionary relationship with all other publicly available DENV-2 genomes in a global and broad temporal context. In this initial tree, we included 3,071 DENV-2 genomic sequences available from the NCBI database and cross-verified these publicly available sequences against other repositories such as Nextstrain [42]. We then pruned out sequences to focus on Nicaraguan sequences and closely related sequences from other countries within the same genotype – the Asian-American genotype. This procedure led to a final set of 358 DENV-2 genomic sequences that was used in both ML and Bayesian phylogenetic analyses. Alignments were produced using MAFFT v7.4 [43] with the ‘globalpair’ algorithm and inspected manually using AliView v1.27 [44]. Maximum likelihood-based phylogenetic inference was conducted using RAxML-HPC Black-Box v8.2 [45] implemented via the CIPRES Science Gateway [46]. The most likely ML tree along with bootstrap support values (based on the automatically chosen number of ML replicates) was visualized using iTOL v6 [47].

Bayesian phylogenetic inference was performed using BEAST v1.10 (Bayesian Evolutionary Analysis for Sampling Trees [48, 49]) with the General Time-Reversible + gamma (GTR + Γ) substitution model, an uncorrelated lognormal molecular clock, and a Bayesian skyline coalescent tree prior. The MCMC (Markov chain Monte Carlo) runs for both the overall phylogeny and the skyline reconstruction included 100,000,000 steps sampled every 1,000 steps, discarding the first 10% of samples (10% burnin). The resulting log files were analyzed in Tracer v1.7. The convergence of the MCMC samples on the posterior distribution was defined at an effective sample size (ESS) value of > 200. The uncertainty in the parameter estimates was assessed using 95% HPD intervals. The maximum clade credibility (MCC) tree was generated using TreeAnnotator v1.10 and visualized using iTOL v6. To reconstruct the DENV demographic history, we employed the Bayesian skyline plot model [50] and generated the Bayesian skyline plot figure using Tracer v1.7.

### Ancestral state reconstruction

To determine the non-synonymous substitutions that characterize novel Nicaraguan DENV-2 lineages, we reconstructed hypothetical sequences of ancestral nodes using the parsimony-based ancestral state reconstruction package in Mesquite v3.61. The nucleotide and amino acid changes were then mapped to the ML tree to identify synapomorphies (shared derived traits) associated with lineages of interest.

The protein structures of pr-E heterodimer (PDB 3C5X), NS1 dimer (PDB 406B), and NS5 (PDB 5JJR) were annotated to show the location of non-synonymous mutations associated with the evolution of NI-3B clade on a DENV-2 genome using the software PyMOL. The figure was made using BioRender.

### Selection pressure analyses

We used GARD (genetic algorithm for recombination detection), implemented via the Datamonkey 2.0 server [51, 52], to first comprehensively screen the complete coding sequences of DENV-2 alignment for recombination breakpoints (none were found). Then, we tested for evidence of natural selection pressure by evaluating the ratio of non-synonymous (dN) to synonymous (dS) mutations (ω ratio) using three methods in HyPhy [53, 54], using p-value cutoffs of 0.05 for statistical significance, implemented in the Datamonkey 2.0 server:

1) Branch-Site Unrestricted Statistical Test for Episodic Diversification (BUSTED) provides a gene-wide test for positive selection. In this analysis, we tested whether the phylogeny of DENV-2 had experienced positive selection at least one site on at least one branch.

2) Adaptive Branch-Site Random Effects Likelihood (aBRESL) tests whether a proportion of sites have evolved under positive selection for each branch in the phylogeny.Significance was assessed using the Likelihood Ratio Test at a threshold of p ≤ 0.05, after correcting for multiple tests.

3) Mixed Effects Model of Evolution (MEME) tests the hypothesis that individual sites have been subjected to episodic positive selection. In other words, MEME aims to detect sites evolving under positive selection in a proportion of branches.

### Electronic supplementary material

Below is the link to the electronic supplementary material.

Supplementary Material 1

## Data Availability

Raw unprocessed sequencing reads are available through the NCBI Sequence Read Archive BioProject PRJNA970723. The assembled whole and partial virus genomes generated in this study have been deposited in GenBank under accession number: OQ782198 - OQ782229.

## Abbreviations

aBRESL: Adaptive Branch-Site Random Effects Likelihood
BEAST: Bayesian Evolutionary Analysis for Sampling Trees
BUSTED: Branch-Site; Unrestricted Statistical Test for Episodic Diversification
CHIKV: Chikungunya virus
Cq: Quantification cycle
DENV: Dengue virus
DENV-2: Dengue virus serotype 2
DF: Dengue fever
DHF/DSS: Dengue hemorrhagic fever/dengue shock syndrome
ESS: Effective sample size
GARD: Genetic Algorithm for Recombination Detection
dN: Non-synonymous mutation
dS: Synonymous mutation
HPD: Highest posterior density
MCC: Maximum Clade Credibility
MCMC: Markov chain Monte Carlo
MEME: Mixed Effects Model of Evolution
ML: Maximum likelihood
MTase: Methyltransferase domain
MRCA: The most recent common ancestor
N_e_: Effective population size
NTC: No-template control
PDCS: the Nicaraguan Pediatric Dengue Cohort Study
RdRp: RNA-dependent RNA polymerase
ZIKV: Zika virus
