# Supplementary material for: Phylodynamics of dengue virus 2 in Nicaragua leading up to the 2019 epidemic reveals a role for lineage turnover
Source: BMC Ecol Evol. 2023 Sep 28;23:58. doi: 10.1186/s12862-023-02156-4 (PMC10537812; doi:10.1186/s12862-023-02156-4)
Supplement: Supplementary file 1 — Supplementary Material 1 [file 12862_2023_2156_MOESM1_ESM.docx]

Supplementary Data

**Phylodynamics of dengue virus 2 in Nicaragua leading up to the 2019 epidemic reveals a role for lineage turnover**

Panpim Thongsripong, Sean V. Edgerton, Sandra Bos, Saira Saborío, Guillermina Kuan, Angel Balmaseda, Eva Harris, Shannon N. Bennett


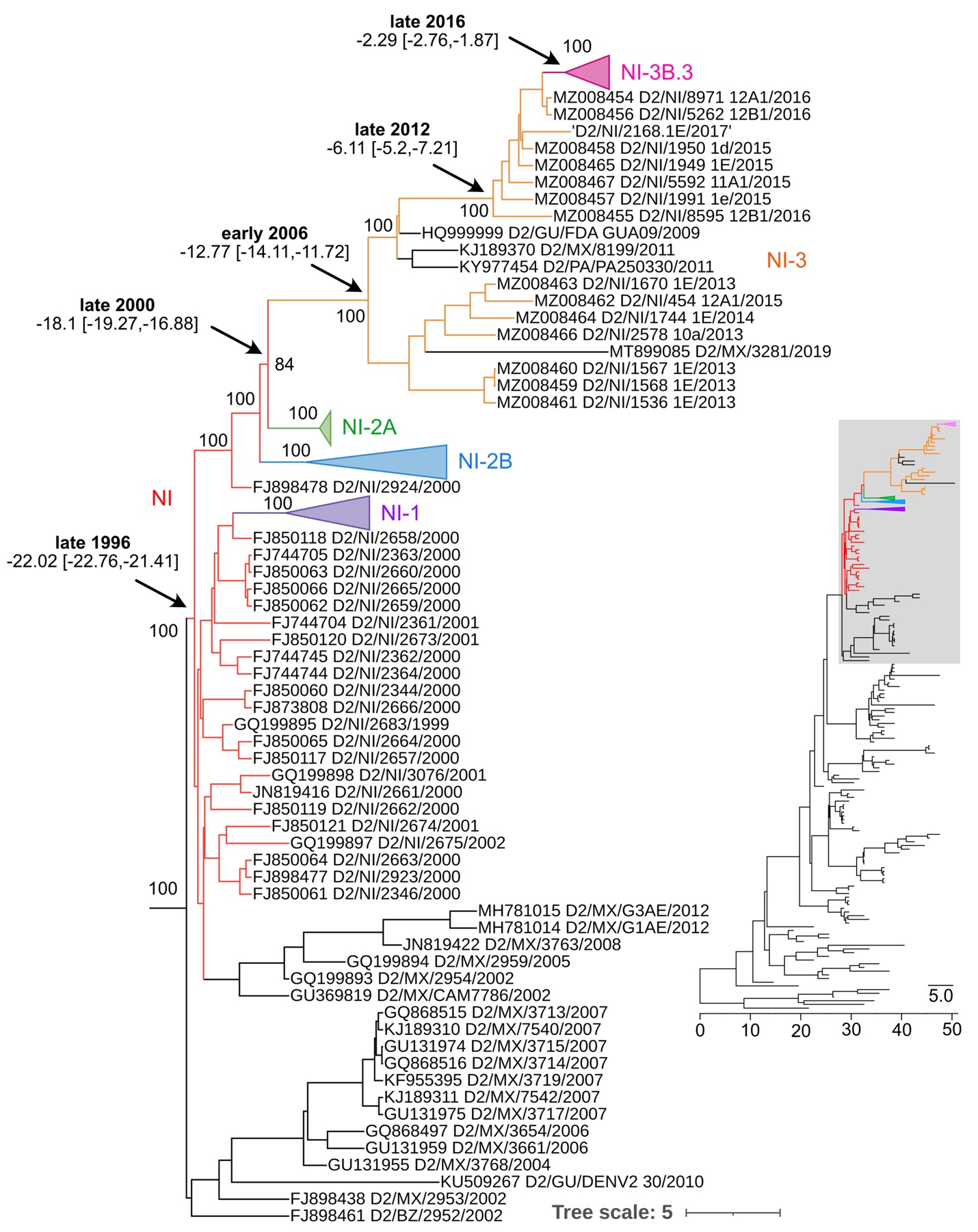


**Supplementary Figure 1.** MCC tree showing node ages and posterior for nodes of interest.

**Supplementary Table 1**. The ω distribution over sites at each significant branch (aBRSEL results).

| **Name** | **Test p-value** | **Number of ω rate categories** | **ω distribution over sites** |
| --- | --- | --- | --- |
| D2/NI/2352/2007 | <0.0001 | 2 | ω_1_=0.393 (100%)  ω_2_=16000 (0.25%) |
| D2/NI/2209/2019 | 0.0001 | 2 | ω_1_=0.638 (100%)  ω_2_=11300 (0.21%) |
| D2/NI/1761/2006 | 0.0001 | 2 | ω_1_=1.00 (100%)  ω_2_=3620 (0.36%) |
| D2/NI/2356/2007 | 0.0003 | 2 | ω_1_=0.0677(100%)  ω_2_=1910 (0.39%) |
| D2/NI/2216/2019 | 0.0026 | 2 | ω_1_=0.00 (100%)  ω_2_=2680 (0.20%) |
| D2/NI/2997/2007 | 0.038 | 1 | ω_1_=10^10^ (100%) |

**Supplementary Table 2.** The inferred aBSREL model complexity of the DENV-2 tree.

| **ω rate classes** | **Number of branches (%)** | **% of tree length** | **Number of branches under selection** |
| --- | --- | --- | --- |
| **1** | 526 (97%) | 84% | 1 |
| **2** | 14 (2.6%) | 16% | 5 |
| **3** | 1 (0.18%) | 0.77% | 0 |
